# Effect of Soft X-ray Irradiation on Film Properties of a Hydrogenated Si-Containing DLC Film

**DOI:** 10.3390/ma14040924

**Published:** 2021-02-15

**Authors:** Kazuhiro Kanda, Ryo Imai, Shotaro Tanaka, Shuto Suzuki, Masahito Niibe, Takayuki Hasegawa, Tsuneo Suzuki, Hiroki Akasaka

**Affiliations:** 1 Laboratory of Advanced Science and Technology for Industry, University of Hyogo, 3-1-2 Koto, Kamigori, Hyogo 678-1205, Japan; szp140@gmail.com (R.I.); shotnr@gmail.com (S.T.); chamomile0409@gmail.com (S.S.); niibe@lasti.u-hyogo.ac.jp (M.N.); t-hasegawa@kmtl.co.jp (T.H.); 2 Department of Nuclear System Safety Engineering, Nagaoka University of Technology, 1603-1 Kamitomioka-machi, Nagaoka, Niigata 940-2188, Japan; suzuki@vos.nagaokaut.ac.jp; 3 Department of Mechanical Engineering, School of Engineering, Tokyo Institute of Technology, 2-12-1 Ookayama, Meguro-ku, Tokyo 152-8550, Japan; akasaka@mech.titech.ac.jp

**Keywords:** diamond-like carbon, Si containing diamond-like carbon film, soft X-ray, synchrotron radiation, X-ray reflectivity, elastic recoil detection analysis, Rutherford backscattering spectrometry, X-ray photoelectron spectroscopy, near-edge X-ray absorption fine structure

## Abstract

The effect of soft X-ray irradiation on hydrogenated silicon-containing diamond-like carbon (Si-DLC) films intended for outer space applications was investigated by using synchrotron radiation (SR). We found that the reduction in film thickness was about 60 nm after 1600 mA·h SR exposure, whereas there was little change in their elemental composition. The reduction in volume was attributable to photoetching caused by SR, unlike the desorption of hydrogen in the case of exposure of hydrogenated DLC (H-DLC) film to soft X-rays. The ratio of the sp^2^ hybridization carbon and sp^3^ hybridization carbon in the hydrogenated Si-DLC films, sp^2^/(sp^2^ + sp^3^) ratio, increased rapidly from ~0.2 to ~0.5 for SR doses of less than 20 mA·h. SR exposure significantly changed the local structure of carbon atoms near the surface of the hydrogenated Si-DLC film. The rate of volume reduction in the irradiated hydrogenated Si-DLC film was 80 times less than that of the H-DLC film. Doping DLC film with Si thus suppresses the volume reduction caused by exposure to soft X-rays.

## 1. Introduction

Amorphous carbon films consisting of mixtures of sp^3^ hybridization carbon and sp^2^ hybridization carbon are known as diamond-like carbon (DLC) films, and DLC films often contain a certain amount of hydrogen [1,2,3]. They have excellent properties such as high hardness, a low friction coefficient, high abrasion quality, a gas barrier, chemical inertness, and surface lubrication [4,5,6,7,8,9,10]. Thanks to these characteristics, DLC films have widespread applications in, for instance, automobile parts, magnetic storage disks, implant parts, and food containers. Expanded use of DLC films has led to studies on their properties. In particular, the last decade has seen the development of novel DLC films that include heterozygous elements, with the exception of carbon and hydrogen [11,12,13,14,15].

Recently, interest has grown in the use of DLC films for outer space applications [16]. In particular, actuators of satellites need lubricants, but the oil that is used in terrestrial applications is unsuitable for outer space because it freezes in a vacuum. DLC films, especially higher hydrogenated DLC (H-DLC) films which contain more than 40% hydrogen, maintain low friction in a vacuum and are regarded as potential solid lubricants in outer space [17,18,19]. The environmental factors in low earth orbit (LEO), which include ultra-high vacuums, soft X-rays, atomic oxygen (AO), and sudden rises and falls in temperature, are very different from those on the ground. To use hydrogenated silicon-containing diamond-like carbon (Si-DLC) film safely in LEO, it is necessary to evaluate its tolerance to other environmental factors.

DLC films have been considered to be durable against X-ray exposure [20]. However, our group has studied the effect of irradiation of H-DLC films by soft X rays and found that: (1) the volume of the H-DLC film is reduced under irradiation by soft X-rays; (2) the main reason for the volume reduction is desorption of hydrogen; and (3) new bonds between carbon atoms are formed by desorption of hydrogen, and the sp^2^/sp^3^ ratio of carbon in the film increases [21,22].

This study investigated irradiation of hydrogenated Si-DLC film by soft X-rays for the purpose of understanding tolerance of films to soft X-rays. Our main interest was in the variations in the volume and properties of the film and the mechanism of these variations. Thereby, we exposed hydrogenated Si-DLC film to soft X-rays using synchrotron radiation (SR) and measured the SR dose dependence of the film thickness, film density, elemental content inside and at the surface of the film, and the local structure of C and Si in the film. We examined the effect of soft X-rays on hydrogenated Si-DLC and the effect of the doping of DLC film with Si.

## 2. Materials and Methods

We paid special attention to the rate and the reaction process involved in the reduction in volume of the hydrogenated Si-DLC film due to irradiation. To investigate the effects of the soft X-ray irradiation inside and on the surface of the hydrogenated Si-DLC films and the influence of Si atoms on these effects, we exposed films to SR in the soft X-ray region in a dose region from 0 to 3000 mA·h and measured various film properties depending on the SR dose as follows: (1) Film thickness, from which the reduction in film volume could be estimated, was measured with a stylus profiler, SEM, and X-ray reflectivity (XRR); (2) Film density was estimated by taking X-ray reflection measurements; (3) The elemental composition of the hydrogenated Si-DLC film was evaluated by a combination of elastic recoil detection analysis (ERDA) and Rutherford backscattering spectrometry (RBS); (4) The surface composition of the hydrogenated Si-DLC film was observed by X-ray photoelectron spectroscopy (XPS); (5) The local structure of carbon and silicon atoms was analyzed on the basis of the C *K* edge, Si *K* edge, and Si *L* edge near-edge X-ray absorption fine structure (NEXAFS) spectra.

### 2.1. Preparation of Samples and SR Irradiation Apparatus

The hydrogenated Si-DLC film was deposited on a Si wafer by using an amplitude-modulated radio-frequency plasma-enhanced chemical vapor deposition method (Nippon ITF Co., Kyoto, Japan) [23]. This method enables DLC films containing a lot of hydrogen to be deposited. The desired thickness of the hydrogenated Si-DLC film was 500 nm. The observed thickness of the as-deposited hydrogenated Si-DLC film was 522 nm using a scanning electron microscope (SEM).

### 2.2. Irradiation of Soft X-rays

The irradiation of the hydrogenated Si-DLC films by soft X-rays was carried out at Beamline 06 (BL06) of the NewSUBARU synchrotron facility of the University of Hyogo located in Kamigori of Japan [24,25]. Details on the experimental apparatus at BL06 have been described in previous reports [26,27]. In brief, the SR extracted from a bending magnet, which is the light source of BL06, was shaped into a straight beam via a pair of mirrors that was incident on the irradiation point. The SR irradiated the sample surface perpendicularly. The SR at the irradiation point had a continuous spectrum from the infrared to soft X-ray region (less than 1 keV). This energy range included 110 eV and 300 eV, which are the ionization energies of the silicon *L* shell and carbon *K* shell, respectively. The penetration depth of 300 eV X-rays was estimated to be 200–300 nm in the DLC film. During the experiment, the electron energy of the NewSUBARU ring was 1.0 GeV and the ring current was kept at 300 mA in the top-up mode. The SR dose (mA·h) was computed as the product of the ring current (mA) and exposure time (h). The hydrogenated Si-DLC film of the sample was fixed to an anoxic copper sample holder and placed at the irradiation point in the irradiation chamber. The temperature of the sample holder was measured using a thermocouple. During the exposure of the hydrogenated Si-DLC films to the soft X-rays, the pressure in the irradiation chamber was on the order of 10^−5^ Pa and the sample holder was confirmed to be at room temperature. In addition, the temperature of the sample holder could be raised to 150 °C by using a heater. After the exposure to SR, the irradiated hydrogenated Si-DLC films were stored in a dry box.

### 2.3. Evaluation of the Reduction in Film Thickness

We measured the film thickness with a stylus profiler to investigate the volume change of the hydrogenated Si-DLC film due to SR irradiation. For this measurement, the hydrogenated Si-DLC films were exposed to SR through an Au mesh mask (wire diameter 0.07 mm R, 100 mesh/inch). The step depth was estimated with the stylus profiler (Bruker, DEKTAK 6M, Billerica, MA, USA), which measured the difference in altitude between the area exposed to SR and the area shadowed by the mesh.

To verify the step-depth measurements, we measured the film thickness by using two other methods, i.e., by observing section images of the hydrogenated Si-DLC films using SEM (JEOL, JSM-6700F, Akishima, Japan) and by estimating the film thickness from the X-ray reflectivity (XRR). The XRR measurement is described in the next section. In both methods, the reduction in film thickness was estimated by subtracting the film thickness after SR irradiation from that of the as-deposited film (522 nm).

### 2.4. X-ray Reflection Measurements

We estimated the densities of the hydrogenated Si-DLC films from the XRR measurements by using an X-ray diffractometer (Phillips, X’Pert PRO MRD, Amsterdam, Nederland). X-ray reflectivity was measured in the range of an incident angle, 2*θ*, from 0.1° to 1.0° by a step of 0.003° by using Cu *K*α as the X-ray source.

### 2.5. Measurements Elastic Recoil Detection Analysis and Rutherford Backscattering Spectrometry

The elemental composition of hydrogenated Si-DLC films was determined by a combination of ERDA and RBS measurements using the electrostatic accelerator (Nisshin High Voltage, NT-1700HS, Kyoto, Japan) at the Extreme Energy-Density Research Institute, Nagaoka University of Technology. The experimental RBS/ERDA apparatus is described in detail in [28,29,30,31]. Briefly, He^+^ ions accelerated to 2.5 MeV using a tandem Pelletron accelerator (High Voltage Engineering Europa, Amsterdam, Nederland) were used as the incident beam, for which the angle with respect to the surface normal was 72°. In the RBS experiments, recoil He^+^ ions were detected using a solid-state detector (SSD) (Ortec, Ortec ULTRA, Oak Ridge, TN, USA), for which the angle with respect to the surface normal was 78°. In the ERDA experiments, hydrogen atoms scattered by He^+^ ions were detected using another SSD, for which the angle with respect to the surface normal was 12°. The observed RBS/ERDA spectra were simulated by a 1% step; therefore, the obtained error of the composition of each element was 1%.

### 2.6. X-ray Photoelectron Spectroscopy Measurements

We estimated the elemental composition of the hydrogenated Si-DLC film surface by using a conventional X-ray photoelectron spectroscopy (XPS) apparatus (Shimadzu, ESCA-1000, Kyoto, Japan). The Mg Kα line was used as the X-ray source, and the incident angle was 45°. The detection depth of XPS using the Mg Kα line (1253.6 eV) was estimated to be less than 3 nm from the surface from the escape depth of photoelectrons.

### 2.7. Measurements of Near-Edge X-ray Absorption Fine Structure

NEXAFS spectroscopy using synchrotron radiation is sensitive to the local structure around the absorber atom [32,33]. To discuss the variations in the local structure of the hydrogenated Si-DLC film due to exposure to SR, the C *K* edge, Si *K* edge, and Si *L* edge NEXAFS spectra were measured. The C *K* edge and Si *L* edge measurements were performed at BL09A in the NewSUBARU synchrotron facility, which has an 11-m-undulator as a light source and a grating monochromator [34,35]. The NEXAFS spectra of the C *K* edge absorption and Si *L* edge absorption were measured using a grating of 1200 and 900 grooves/mm in the ranges 275–330 eV and 100–125 eV, respectively. The energy resolution was estimated to be less than 0.5 eV (FWHM). The NEXAFS measurement of the Si *K* absorption was performed at BL05A of the NewSUBARU synchrotron facility, which has a bending magnet as a light source and a double crystal monochromator [36]. The NEXAFS spectra of the Si *K* edge absorption were measured in the range 1810–1890 eV using InSb(111). The energy resolution was estimated to be less than 2.0 eV (FWHM). In the measurement at both beamlines, the irradiation angle at the sample was the “magic angle” of 54.7° with respect to the surface. The electrons coming from the sample were detected in total electron yield (TEY) mode. The intensity of the incident photon beams (I_0_) was measured by monitoring the photocurrent from a gold mesh. The absorption signal was given by the ratio between the out-coming electron intensity from the sample, I_s_, and I_0_. The detection depth of the NEXAFS measurement in TEY mode was reported to be about 4 nm at the N *K* edge of 409 eV [37].

## 3. Results

### 3.1. Reduction in Thickness of Hydrogenated Si-DLC Film

**Figure 1:**
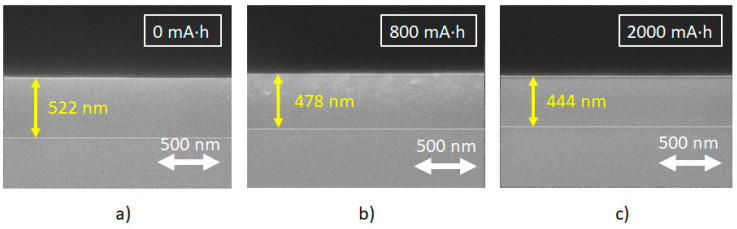
Cross-section of the hydrogenated silicon-containing diamond-like carbon (Si-DLC) films (**a**) before irradiation, and after irradiation of (**b**) 800 mA·h and (**c**) 2000 mA·h, observed using SEM.

The reduction of the film volume is undoubtedly important for the utilization of the hydrogenated Si-DLC film. As mentioned in Section 2.3., we observed the change in film thickness in three different ways. Examples of cross-sectional observation of the hydrogenated Si-DLC films by SEM are shown in Figure 1.

**Figure 2:**
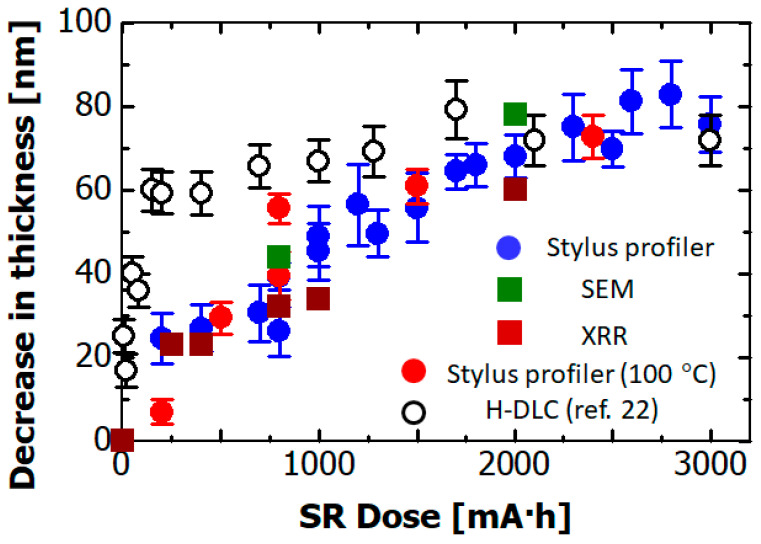
Synchrotron radiation (SR) dose dependence of the reduction in film thickness of the hydrogenated Si-DLC film measured with a stylus profiler (blue circles), SEM (green squares), and X-ray reflectivity (XRR, brown squares). Red circles indicate reductions in film thickness acquired by the stylus profiler in case of substrate heated to 100 °C with a heating system. Open circles indicate reductions in the film thickness of non-doped hydrogenated DLC (H-DLC) film after SR exposure [22].

The SR dose dependence of the decrease in thickness of the hydrogenated Si-DLC film is depicted in Figure 2. The blue circles show the reduction in thicknesses measured using the stylus profiler. The green and brown squares show the reduction in thicknesses estimated from the section images of films before and after SR irradiation and from XRR, respectively. The reduction estimated by the stylus profiler was in good accordance with those estimated from the SEM and XRR measurements. The reduction gradually reached about 60 nm after 2000 mA·h of SR exposure. It remained constant at doses greater than 2000 mA·h. These results show that by doping H-DLC with Si, the effect of SR radiation was lower, and only at higher X-ray doses did the effect become comparable for the two kinds of materials.

In addition, we exposed the hydrogenated Si-DLC film to SR while keeping the substrate temperature at 100 °C by using a heating system. The red circles show the reduction in film thickness measured using the stylus profiler when the substrate was heated. The rate of reduction did not change when the substrate temperature rose. This result, in which reaction rate did not depend on the substrate temperature, indicates that the reduction in film thickness does not proceed via a process ruled by activation energy.

The open circles show the reduction in film thickness of H-DLC film (in which no element was doped) due to SR exposure, the values of which were converted from the film thicknesses reported in Reference [22]. The reduction in film thickness was about 60 nm after 20 mA·h SR exposure. On the other hand, in the case of SR irradiation of the hydrogenated Si-DLC film, a dose of 1600 mA·h was needed to cause a reduction in film thickness of 60 nm. Namely, the rate of reduction in volume was 80 times lower that of the non-doped hydrogenated DLC film. As a result, it was found that doping hydrogenated DLC film with Si reduced the rate of reduction in film thickness due to SR exposure.

### 3.2. Film Density

**Figure 3:**
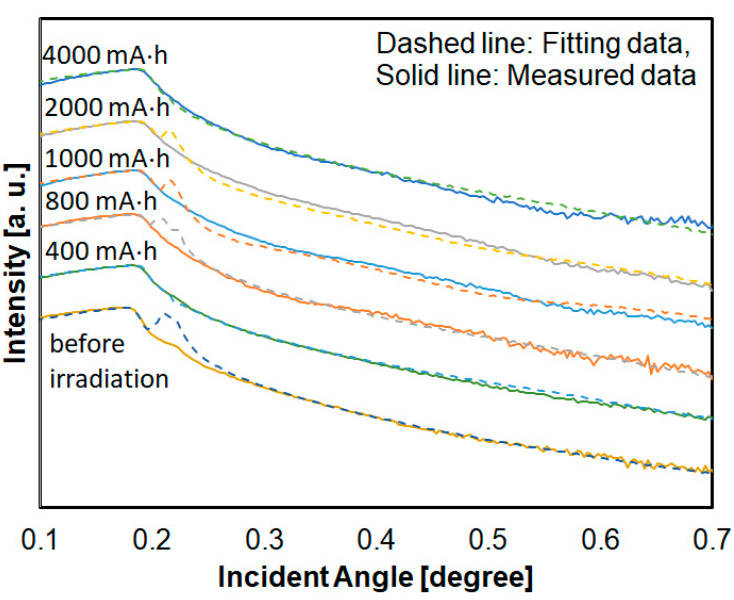
XRR profiles and fitting results of hydrogenated Si-DLC film.

Film density is an important index to distinguish the volume change of film due to photoetching or shrinkage. Since it is thought that these densities have a distribution in the thickness direction, there were some samples that could not completely satisfy the fitting, especially at an angle slightly higher than the critical angle. Some samples also could not be completely fitted by single Si-DLC layer model. Therefore, profiles of Si-DLC films were fitted by a one-layer model as much as possible around the critical angle. Figure 3 shows the XRR profiles and fitting results. XRR analyses were performed using X’ Pert Reflectivity software (Phillips, Amsterdam, Nederland).

**Figure 4:**
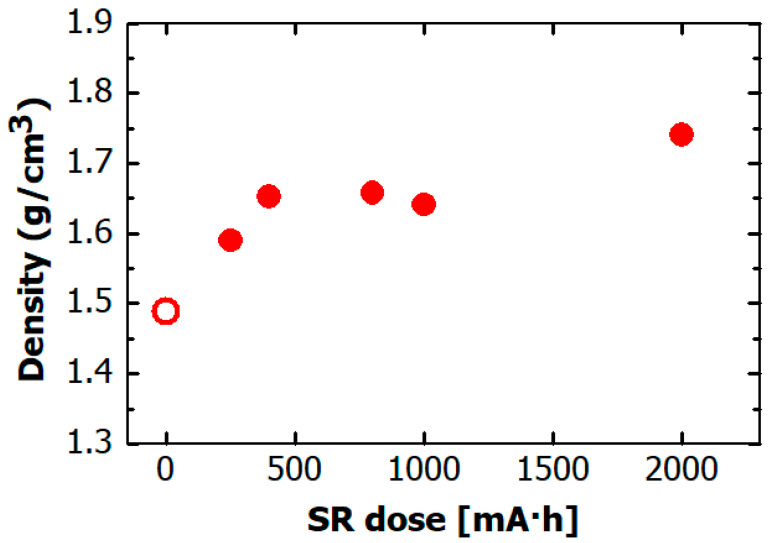
Dependence on SR dose of density of hydrogenated Si-DLC film. The open circle indicates density of hydrogenated Si-DLC film before SR irradiation.

Figure 4 shows the film density dependence on SR dose obtained from the XRR measurements. The value in this figure describe the density of the thickest layer of these layers. The density of the hydrogenated Si-DLC film before irradiation was ~1.5 g/cm^3^. It markedly increased to ~1.65 g/cm^3^ after 500 mA·h of SR exposure, and remained constant at 1.65 g/cm^3^ at doses of more than 500 mA·h. These results indicate that the increase in film density caused by the SR exposure ceased at a small SR dose within 500 mA·h, which is different from the case of irradiation of non-doped H-DLC film by soft X-rays, where the film density increased after more than 1500 mA·h of SR exposure [22].

### 3.3. Elemental Composition of Hydrogenated Si-DLC Film

**Figure 5:**
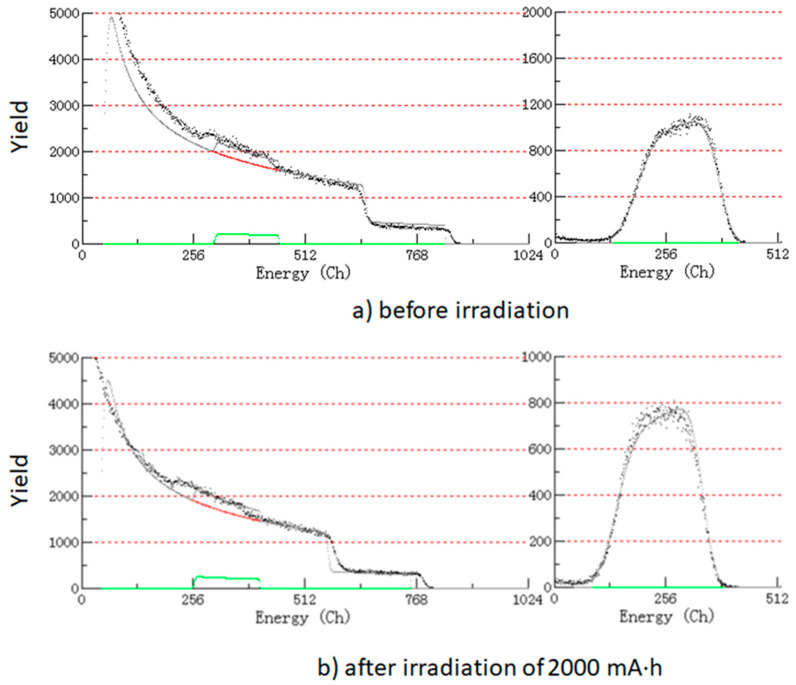
Rutherford backscattering spectrometry (RBS) profile (left side) and elastic recoil detection analysis (ERDA) profile (right side) of (**a**) Si-DLC film before irradiation and (**b**) Si-DLC film after irradiation of 2000 mA·h.

As described in the introduction, the exposure to SR causes the volume of the H-DLC film to shrink because of the desorption of hydrogen [21,22]. Therefore, the dose dependence of the hydrogen content is the most notable factor when discussing the effect of SR on hydrogenated Si-DLC films. The elemental composition of hydrogenated Si-DLC films was determined by a combination of RBS and ERDA measurements. The RBS and ERDA profiles of Si-DLC films before irradiation of SR (Figure 5a) and after irradiation of 2000 mA·h (Figure 5b) are shown as representative examples.

**Figure 6:**
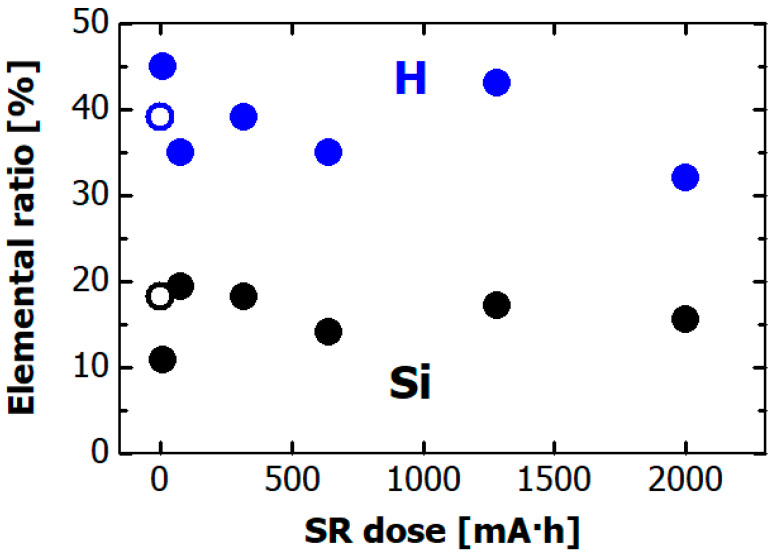
Dependence on SR dose of elemental composition ratio in hydrogenated Si-DLC film, as estimated by ERDA and RBS. Blue and black circles respectively indicate hydrogen and silicon ratios. The open circle indicates hydrogen and silicon ratios before the SR irradiation.

The dose dependences of the hydrogen and silicon composition ratios in the hydrogenated Si-DLC films are shown in Figure 6. The atomic ratios were ~0.4 for hydrogen and ~0.2 for silicon before the SR irradiation (open circles in the figure). Other elements were not found in the RBS spectrum. Moreover, the hydrogen and silicon composition ratios did not change after SR exposure. This result means that desorption of hydrogen from the hydrogenated Si-DLC film was not caused by the exposure to soft X-rays. Namely, doping the DLC film with Si helps to suppress desorption of hydrogen from film.

### 3.4. Elemental Composition and Chemical State of Hydrogenated Si-DLC Film Surface Acquired by X-ray Photoelectron Spectroscopy

**Figure 7:**
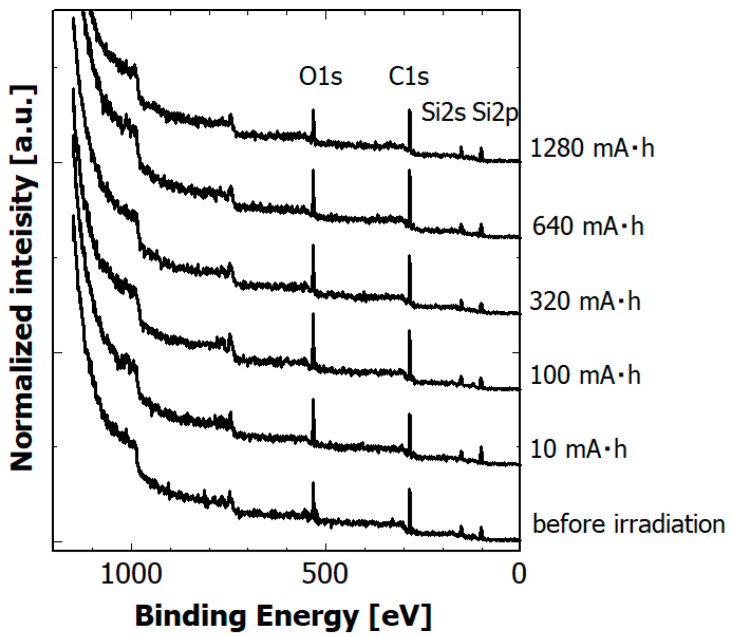
Dependence of XPS spectra of hydrogenated Si-DLC films on SR dose.

Surface modification by exposure to soft X-rays often changes the elemental composition of the surface due to selective photon-stimulated desorption and/or surface oxidation. Figure 7 shows the dependence of XPS spectra of hydrogenated Si-DLC films on SR dose.

**Figure 8:**
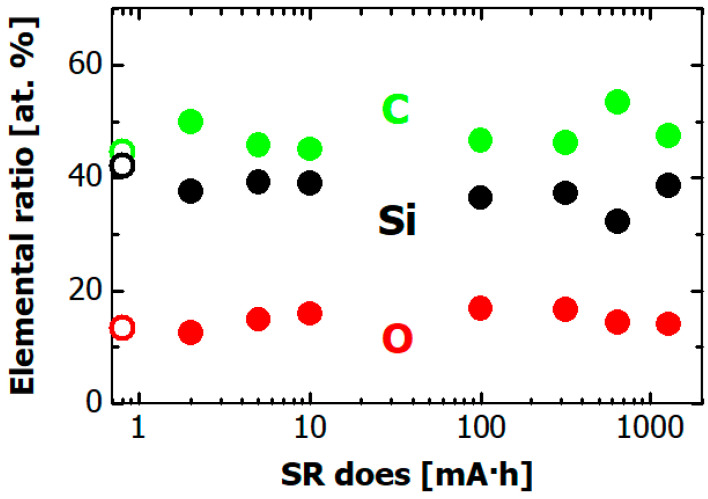
Dependence on SR dose of the elemental ratio of hydrogenated Si-DLC film surface as measured by XPS. Black, green, and red circles respectively show silicon, carbon, and oxygen contents. The open circles indicate carbon, silicon, and oxygen contents before the SR irradiation.

The dose dependences of the atomic composition of the hydrogenated Si-DLC film surface are plotted in Figure 8. The black, green, and red circles respectively show silicon, carbon, and oxygen content. The silicon, carbon, and oxygen contents before irradiation (open symbols in the figure) were ~0.4, ~0.4, and ~0.2, respectively. The observed oxygen is ascribable to surface oxidation rather than contamination during the film deposition because oxygen was not observed in the RBS spectra. Other elements were not found in the XPS spectrum before nor after SR irradiation. The atomic composition of the film surface was not changed after the SR exposure. Note that hydrogen content could not be measured in this XPS measurement.

### 3.5. Local Structure of Carbon and Silicon Atoms in Hydrogenated Si-DLC Film

**Figure 9:**
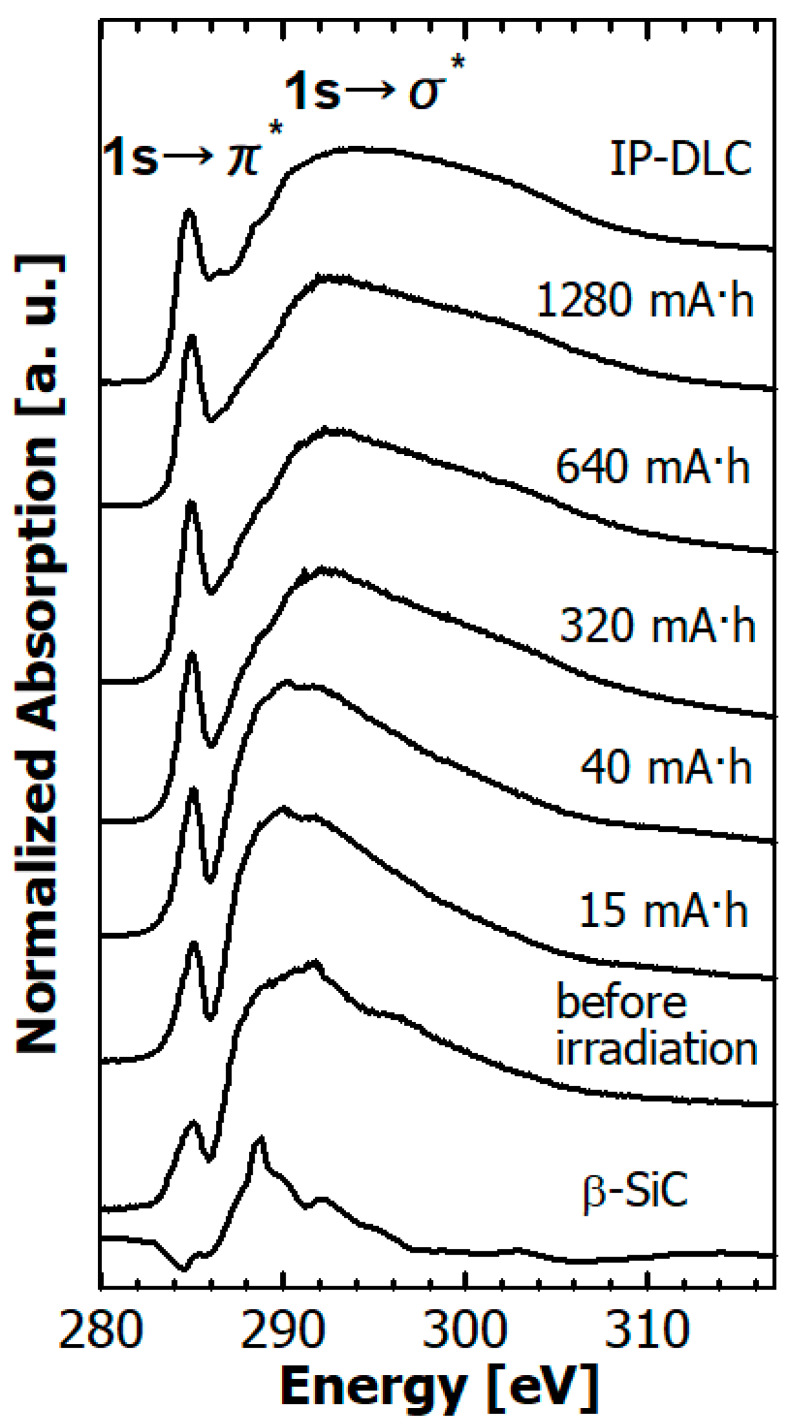
Dependence of the C *K* edge near-edge X-ray absorption fine structure (NEXAFS) spectra of hydrogenated Si-DLC films on SR dose, together with those of β-SiC powder and IP-DLC film.

The C *K* edge NEXAFS spectra of the hydrogenated Si-DLC film before and after SR irradiation are depicted in Figure 9 together with those of β-SiC powder and typical commercial DLC film. The commercial film was deposited on 200-nm-thick Si wafers using the ion plating method, referred to as IP-DLC film hereafter. The IP-DLC film consisted of only carbon and hydrogen without silicon or other hetero-elements. The hydrogen content of the IP-DLC film was estimated to be ~20% from a combination of ERDA and RBS measurements.

The sharp π* peak observed at 285.4 eV is ascribed to the C 1s→π* resonance transition originating from the carbon–carbon double bond. The broad σ* peak observed at about 285–310 eV is ascribed to the C1s → σ* resonance transition [38]. The C *K* edge NEXAFS spectra of the Si-DLC films were classified into four types according to silicon content [39]. The C *K* edge NEXAFS spectra of the hydrogenated Si-DLC films before SR irradiation were classified as type 3 reported in Reference [39]. That is, the σ* peak was narrower than that of typical DLC film and shifted to the lower energy of ~5 eV due to the influence of the peak at 289 eV derived from the 1s → σ*(C-Si) transition. In addition, the peak intensity of the 1s → π* transition at 285.4 eV was weak.

The features of the C *K* edge NEXAFS spectra of hydrogenated Si-DLC film greatly varied between before and after irradiation by soft X-rays. The peak intensity of the 1s → π* transition at 285.4 eV increased with an SR dose of 15 mA·h, and it increased gently with an SR dose greater than 15 mA·h. The σ* peak became broader and the intensity peak shifted to a higher energy. In other words, the C *K* edge NEXAFS spectra of the hydrogenated Si-DLC films changed from type 3 to type 1 or 2. As a result, the spectral features of the hydrogenated Si-DLC film after SR exposure of more than 320 mA·h resembled those of typical DLC film consisting only of carbon and hydrogen without silicon.

**Figure 10:**
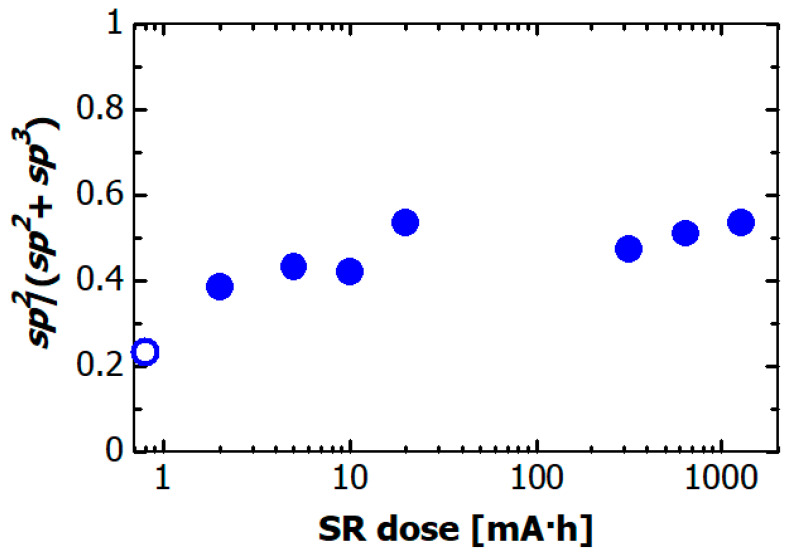
Dependence of the sp^2^/(sp^2^ + sp^3^) ratio in hydrogenated Si-DLC film on the SR dose. The open circle indicates sp^2^/(sp^2^ + sp^3^) ratio of hydrogenated Si-DLC film before SR irradiation.

The C atoms in the DLC films consist of sp^2^ hybridization carbon and sp^3^ hybridization carbon. The sp^2^/sp^3^ ratio is considered to be a structural parameter that characterizes DLC film properties [3,40]. The absolute sp^2^/(sp^2^ + sp^3^) ratio of carbon atoms in the DLC film can be accurately determined from the C *K* edge NEXAFS spectra, as described in Refs. [41,42,43]. The SR dose dependence of the sp^2^/(sp^2^ + sp^3^) ratio in the hydrogenated Si-DLC film is plotted in Figure 10. The amount of sp^2^ bonded carbon atoms can be determined by normalizing the area of the resonance corresponding to the 1s → π* transitions at 285.4 eV with a large section of the spectrum. The absolute sp^2^/(sp^2^ + sp^3^) ratio was determined by comparing the normalized 1s → π* area from the sample with that from the NEXAFS spectrum of highly ordered pyrolytic graphite (HOPG) as a standard sample. The sp^2^/(sp^2^ + sp^3^) ratio of the hydrogenated Si-DLC film before irradiation (open circle) was ~0.23, which indicated that sp^3^ hybridized carbon was a major component of the hydrogenated Si-DLC film. The sp^2^/(sp^2^ + sp^3^) ratio increased rapidly to ~0.5 for SR doses less than 20 mA·h. At doses of more than 20 mA·h, the sp^2^/(sp^2^ + sp^3^) ratio remained approximately constant at ~0.5.

**Figure 11:**
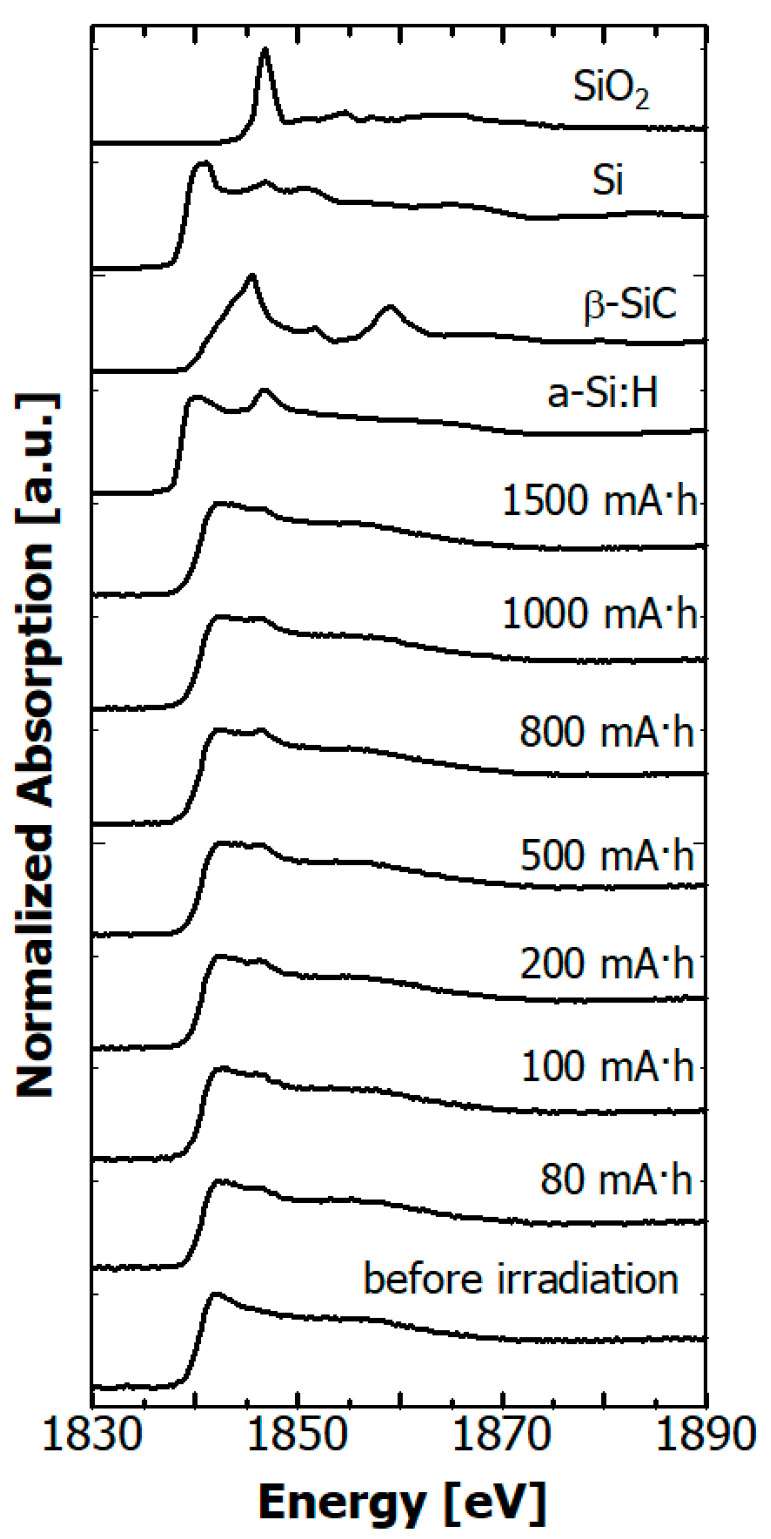
Dependence of Si *K* edge NEXAFS spectra of hydrogenated Si-DLC films on SR dose, together with those of SiO_2_ powder, Si wafer, β-SiC powder, and a-Si:H film.

The Si *K* edge NEXAFS spectra of the hydrogenated Si-DLC film before after irradiation are depicted in Figure 11 together those of reference materials, SiO_2_ powder, Si wafer, β-SiC powder, and amorphous hydrogenated silicon (a-Si:H) film. In the spectra of the Si wafer and a-Si:H film, peaks from SiO_2_ due to native oxidation were observed at 1846.8 eV [44]. Si atoms in the hydrogenated Si-DLC film can be expected to have Si, C, H, and/or O as neighboring atoms. The chemical environment around the absorbing atom can be evaluated from the positions of the absorption edge and the white line in the NEXAFS spectrum. The Si *K* edge shifts towards the higher energy side with increasing positive charge on the Si atom [45,46]. The edge from the hydrogenated Si-DLC film before irradiation was at ~1840 eV, i.e., near that of the Si wafer and a-Si:H film. The white line was observed at about 3 eV above the absorption edge, which resembled that of the Si wafer and a-Si:H film. These results indicate that the chemical environment of Si atoms in the hydrogenated Si-DLC film was similar to that in the Si wafer and a-Si:H film. After SR irradiation, a peak at 1846.8 eV, which was derived from SiO_2_, appeared in the NEXAFS spectra of the Si-DLC films. The intensity reached a maximum at 800 mA·h and decreased beyond 1000 mA·h. On the other hand, the intensity increased in the energy range of about 1843–1846 eV. The peak in this energy range was reported to be due to deoxidization of SiO_2_ [45]. These results mean that an oxidized Si layer was generated at the surface of the hydrogenated Si-DLC film by oxidation of surface Si atoms with residual oxygen in the vacuum chamber due to SR irradiation. The oxidized Si layer was deoxidized by additional exposure to soft X-rays. On the other hand, the SR exposure did not change the positions of the absorption edge and the white line. Therefore, the chemical environment around the Si atoms in the hydrogenated Si-DLC film was not changed by the SR irradiation.

**Figure 12:**
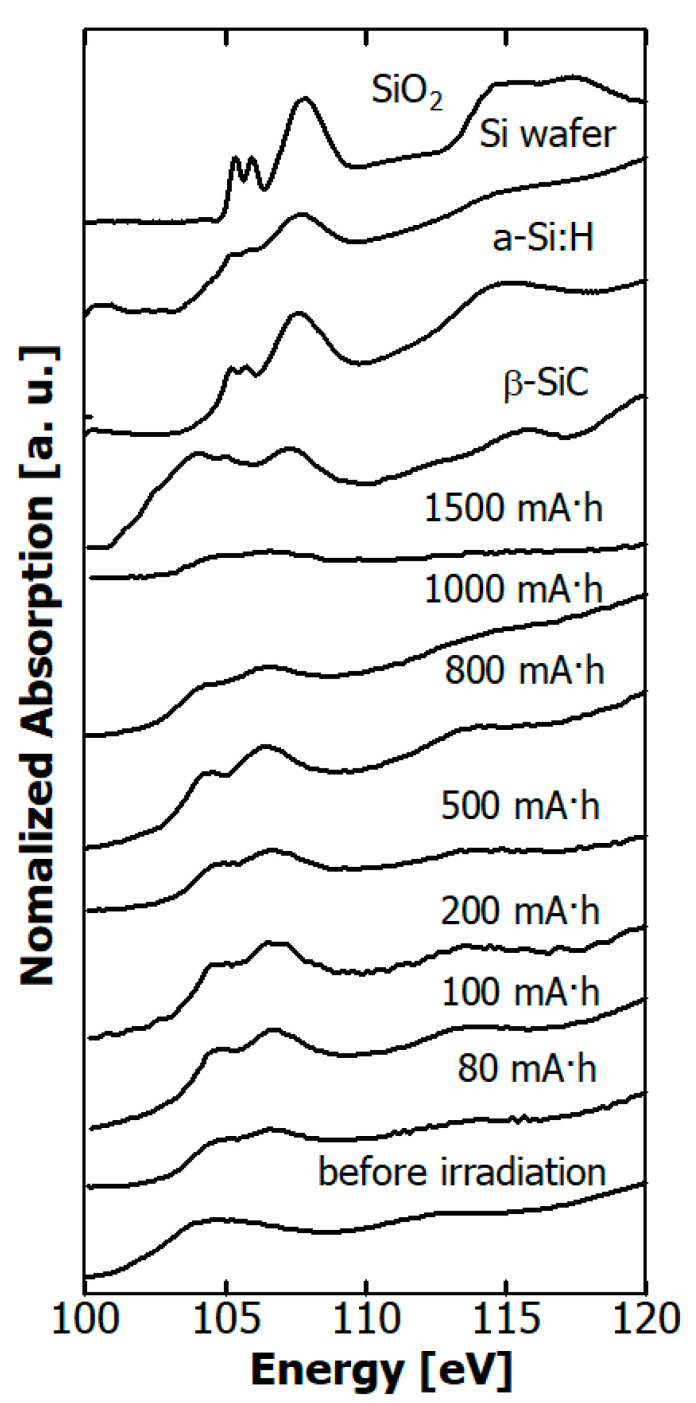
Dependence of Si *L* edge NEXAFS spectra of hydrogenated Si-DLC films on SR dose, together those of SiO_2_ powder, Si wafer, a-Si:H film, and β-SiC powder.

The Si-*L* edge NEXAFS spectra of the hydrogenated Si-DLC film before and after irradiation are depicted in Figure 12 together with those of SiO_2_ powder, Si wafer, a-Si:H film, and β-SiC powder. In the spectra of the Si wafer and a-Si:H film, peaks derived from SiO_2_ due to native oxidation were observed at 106 eV [44]. In the range of 0–800 mA·h, the intensity of the peak arising from SiO_2_ increased, while it decreased at doses greater than 1000 mA·h, as in the Si *K* edge spectra.

## 4. Discussion

The effect of soft X-rays irradiating hydrogenated Si-DLC films was investigated for dose ranges up to 3000 mA·h. The thickness (film volume) of hydrogenated Si-DLC film reduced after the exposure to continuous soft X-rays up to 1000 eV. However, the rate of reduction in film thickness of the hydrogenated Si-DLC film was much lower than that of the H-DLC film. The density of the hydrogenated Si-DLC film rose a little as a result of the exposure to soft X-rays. The elemental composition (H, C, and Si) of the hydrogenated Si-DLC film, which was estimated from the ERDA/RBS study, did not significantly change as a result of the exposure. In addition, no significant change occurred in the surface elemental composition (C, Si, and O) in the XPS measurement. On the other hand, C *K* edge NEXAFS measurements showed that soft X-ray exposure significantly changed the local structure of the C atoms, while the Si *K* edge and Si *L* edge NEXAFS measurements showed no significance.

From these experimental results, the reason why the rate of decrease in film thickness of hydrogenated Si-DLC film is much smaller than that of H-DLC film is ascribable to the process of the reduction in film thickness. The dominant process in the exposure of H-DLC film to the soft X-rays is shrinkage of the film by desorption of hydrogen from inside the film. These changes in the C-*K* edge NEXAFS spectra are believed to correspond to hydrogen desorption from the film. As described in Reference [21], hydrogen content in H-DLC films decreases from ~50% to ~30% due to 200 mA·h SR exposure. On the other hand, in the exposure of the hydrogenated Si-DLC film to soft X-rays, hydrogen does not desorb from the interior of the film. In this case, the dominant process of the decrease in thickness is photoetching, which is much slower than the shrinkage due to desorption of hydrogen. This photoetching process is not one in which the activation energy determines the reaction rate, because the rate of decrease in film thickness does not change when the substrate temperature rises. In other words, this photoetching does not proceed through a temperature rise in a local area due to exposure to SR, but rather through direct inner shell excitation of the C and Si atoms in the hydrogenated Si-DLC film by the soft X-rays.

Soft X-rays caused reactions several nm from the surface of the hydrogenated Si-DLC film. In the C *K* edge NEXAFS spectra, the intensity of the 1s → π* transitions at 285.4 eV increased and the peak of the 1s → σ* transition shifted with increasing SR dose to the high energy side in the dose range less than 20 mA·h. The shift of the peak of 1s → σ* transition was ascribable to the reduction in C atoms coupling with Si and H atoms. The peak of C—Si was observed at ~287 eV [47], and the peak at 287.8 eV was assigned to the 1s → σ*(C—H) transition [33,38]. Therefore, the C—Si and C—H bonds at the surface of the hydrogenated Si-DLC films decreased as a result of soft X-ray exposure. The sp^2^/(sp^2^ + sp^3^) ratio of the C atoms increased from 0.23 to 0.5 in the dose range up to 20 mA·h. An increase in the sp^2^/(sp^2^ + sp^3^) ratio due to irradiation by soft X-rays was also observed in the irradiated H-DLC film, and this increase was concluded to occur through formation of C-C double bonds induced by the coupling between C atoms through desorption of hydrogen [22]. The change in the sp^2^/(sp^2^ + sp^3^) ratio of C atoms is caused by irradiation of the hydrogenated Si-DLC film is considered to be due to the same process as in the irradiation of the H-DLC film. The structural change affecting the C atoms occurs only in a very thin surface layer of the hydrogenated Si-DLC film, because the rate of increase in the sp^2^/(sp^2^ + sp^3^) ratio ceased for SR doses greater than 20 mA·h.

O atoms existed on the surface of the hydrogenated Si-DLC film before it was exposed to soft X-rays. The O atoms adsorbed to the surface of the films during storage in the atmosphere rather than during the film deposition, because the ERDA study did not show any O atoms inside the film. The peak of the Si coupling with O atoms was observed in both the Si *K* edge and *L* edge NEXAFS spectra after the films were exposed to soft X-rays. On the other hand, the peak of the C atoms neighboring O atoms at 288.8 eV, which was assigned to the 1s → π* (carboxyl group) [48], did not appear in the C *K* edge NEXAFS spectra. This peak was observed in spectra of surface oxidized DLC film [49]. Therefore, O atoms are more likely to couple with Si atoms than C atoms. Irradiation by soft X-rays caused the O adhering to the surface to combine with Si, but further irradiation caused de-oxidation.

As described above, it was found that the volume change of the hydrogenated DLC film due to soft X-ray irradiation was suppressed by silicon doping into film. This is attributed to the suppression of hydrogen desorption from the film. We believe that the mechanism of the suppression of hydrogen desorption by silicon doping may be as follows: (1) Si atoms on the surface of the hydrogenated Si-DLC film are spontaneously oxidized to silicon oxide, which suppresses hydrogen desorption from inside the film; (2) Soft X-ray irradiation desorbs hydrogen from the film near the surface, increasing the concentration of Si atoms in the surface vicinity, which oxidizes in the same way as in (1), and the silicon oxide layer suppresses hydrogen desorption; and (3) Soft X-ray irradiation desorbs hydrogen from the film near the surface, forming the C-Si network in the surface vicinity, which suppresses hydrogen desorption. We are planning to conduct experiments to test these hypotheses.

## 5. Conclusions

The effect of SR on the hydrogenated Si-DLC film was investigated by continuous soft X-ray irradiation at less than 1000 eV, which is an important environmental factor in LEO. The dependences of the volume, elemental composition, and local structure of C and Si atoms on the SR dose were measured in a dose range up to 3000 mA·h. The reduction in volume of the H-DLC film by the exposure to soft X-rays was due to the bonds between C and H being broken and hydrogen being desorbed, with the remaining C being combined with other C atoms in the DLC film. These processes could be suppressed by doping the film with Si. The dominant process affecting the volume reduction of the hydrogenated Si-DLC film due to the exposure to soft X-rays was photoetching and the rate of reduction was 80 times slower than that of the H-DLC film. The irradiated film was modified only within the vicinity (several nm) of the surface. In addition, it was observed that Si and absorbed O were combined due to exposure to soft X-rays and separated due to excessive soft X-ray irradiation in most surface.

H-DLC films have a low coefficient of friction in vacuum, but their lifetime is short due to desorption of hydrogen during friction. The results of this study indicate the possibility of retaining H in the film by reacting with Si present in the film, which is expected to extend the life of H-DLC film. Further investigations will be needed before hydrogenated Si-DLC films can be used on LEO satellites. We are planning to perform experiments on (1) the dependence of film durability against X-rays versus Si content of the film; (2) simultaneous irradiation of samples with X-rays and AO; and (3) lubricant performance.

## Data Availability

Data sharing not applicable.
